# Simultaneous synthesis of nanodiamonds and graphene via plasma enhanced chemical vapor deposition (MW PE-CVD) on copper

**DOI:** 10.1186/s40064-016-2201-x

**Published:** 2016-05-10

**Authors:** Steven Gottlieb, Nicolas Wöhrl, Stephan Schulz, Volker Buck

**Affiliations:** Faculty of Physics and CENIDE, University Duisburg Essen, Carl-Benz-Straße 199, 47057 Duisburg, Germany; Faculty of Chemistry and CENIDE, University Duisburg-Essen, Carl-Benz-Straße 199, 47057 Duisburg, Germany

**Keywords:** Graphene, Nanodiamond, Hybrid, PE-CVD, Plasma, Raman, OES

## Abstract

The simultaneous growth of both nanodiamonds and graphene on copper samples is described for the first time. A PE-CVD process is used to synthesize graphene layers and nanodiamond clusters from a hydrogen/methane gas mixture as it is typically done successfully in thermal CVD processes for graphene synthesis. However, the standard thermal CVD process is not without problems since the deposition of graphene is affected by the evaporation of a notable amount of copper caused by the slow temperature increase typical for thermal CVD resulting in a long process time. In sharp contrast, the synthesis of graphene by PE-CVD can circumvent this problem by substantially shortening the process time at holding out the prospect of a lower substrate temperature. The reduced thermal load and the possibility to industrially scale-up the PE-CVD process makes it a very attractive alternative to the thermal CVD process with respect to the graphene production in the future. Nanodiamonds are synthesized in PE-CVD reactors for a long time because these processes offer a high degree of control over the film’s nanostructure and simultaneously providing a significant high deposition rate. To model the co-deposition process, the three relevant macroscopic parameters (pressure, gas mixture and microwave power) are correlated with three relevant process properties (plasma ball size, substrate temperature and C_2_/H_α_-ratio) and the influence on the quality of the deposited carbon allotropes is investigated. For the evaluation of the graphene as well as the nanodiamond quality, Raman spectroscopy used whereas the plasma properties are measured by optical methods. It is found that the diamond nucleation can be influenced by the C_2_/H_α_-ratio in the plasma, while the graphene quality remains mostly unchanged by this parameter. Moreover it is derived from the experimental data that the direct plasma contact with the copper surface is beneficial for the nucleation of the diamond while the growth and quality of the graphene benefits from a larger distance to the plasma. Therefore, this work presents a basis for a method to tailor the deposition of graphene–diamond hybrid films using a MW PE-CVD process or to suppress the diamond deposition entirely if desired.

## Background

Graphene as well as nanodiamonds, both allotropes of carbon, have obtained significant interest in the last couple of years from scientific as well as from industrial point of view due to their remarkable properties.

The regular hexagonal pattern in a crystalline monolayer of sp^2^-bonded carbon atoms is the reason for the remarkable mechanical, electronic, thermal, optical and chemical properties of graphene. Optical transparence of up to 97 % and electron mobility above 15,000 cm^2^/Vs have been reported (Wang et al. 2010). The theoretical limits are estimated to be as high as 200,000 cm^2^/Vs for free-standing graphene (Akturk and Goldsman 2008) limited by the scattering of graphene’s acoustic phonons. Due to these properties graphene receives much attention from fundamental as well as applied science and technology. However, the difference between theoretically possible values and the actually achieved ones originate from defects in the graphene sheet and interactions with the underlying substrate.

The first method to isolate graphene sheets was the method of mechanical exfoliation using Scotch tape to pull apart the layers of sample of highly oriented pyrolytic graphite and subsequently transfer single layers onto a SiO_2_ substrate (Novoselov et al. 2004). This method was also used by the Nobel price laureates K. S. Novoselov and A. K. Geim. As would seem natural, this approach is slow and the obtained graphene sheet is limited in size which results in the need for a production route suitable for industrial production.

One of the most frequently used methods to synthesize larger areas of high quality graphene is the use of CVD, in particular thermal CVD, which allows to deposit rather large areas with graphene of high quality (Reina et al. 2009). During the low-pressure CVD process, reactive gas species (mostly H_2_ and CH_4_) are fed into a hot-wall reactor at temperatures of around 1000 °C to initialize chemical reactions. Most of these CVD processes use copper substrates and take advantage of the catalytic influences of the copper on the dissociation of hydrogen (Li et al. 2009; Vlassiouk et al. 2011). However, the high activation temperature for the catalytic reaction of methane causes significant evaporation of the substrate material even at temperatures far below the melting point of copper (Li et al. 2009) resulting in defects in the growing graphene monolayer. In combination with the slow increase of temperature in a typical thermal CVD reactor leading to a long process time, the process will be affected by the evaporation of a notable amount of copper.

Synthesis of graphene by PE-CVD is promising to reduce the problem of evaporation by substantially shortening the process time and having the lowering of the substrate temperature in prospect. The reduced thermal load and the possibility to industrially scale-up the PE-CVD process makes it a very attractive alternative to the thermal CVD process giving hope to synthesize a defect-free graphene layer on larger scale, which is essential for its industrial use such as energy applications (batteries, fuel cells) and semiconductor technology.

There have been some reports of plasma-based methods to decrease the process temperature including the use of microwave plasma CVD to synthesize graphene on nickel foil (Kim et al. 2011a), surface wave plasma CVD to synthesize graphene at temperatures in the range of 300–400 °C on large area conductive electrodes (Kim et al. 2011b; Kalita et al. 2012) and the plasma-assisted deposition of graphene on copper foils at temperatures down to 600 °C (Chan et al. 2013).

Recently a fast, versatile and reproducible PE-CVD method for the synthesis of SLG on large areas has been presented. It was shown that SLG sheets of several mm^2^ were deposited on copper substrates from the PE-CVD process (Woehrl et al. 2014).

However, during the PE-CVD process it was discovered that under certain process conditions a co-deposition of graphene and nanodiamond crystals could be observed on the copper surface.

Usually ultra-nanocrystalline diamond (UNCD) films with grain sizes between 5 and 100 nm are deposited from argon-rich CH_4_/H_2_ plasmas (Woehrl and Buck 2007; Buck and Woehrl 2008). In these processes it could be observed by the mean of plasma characterization (optical emission spectroscopy and mass spectroscopy) that by controlling the C_2_/H_α_ ratio in the plasma it is possible to adjust the nucleation density and the growth rate of the deposited films. It was discovered that the nucleation density increases with the C_2_/H_α_ intensity while the size of the individual diamond crystals decrease at the same time (Woehrl and Buck 2011).

In this work the influence of the plasma parameters (size of plasma ball, C_2_/H_α_ ratio) on the growth process of these two carbon allotropes is investigated. It is aimed to control the deposition of graphene and nanodiamonds independently on each other. Special focus is on process parameters that enable the control of carbon phases formed on the surface to control the allotropes that are synthesized independently and to understand the processes that lead to the respective structures.

## Methods

### Synthesis

The co-deposition of Graphene and nanocrystalline diamond structures was performed with a 2.45 GHz IPLAS *CYRANNUS*^**®**^ I-6′′ plasma source with a maximum of 5 kW microwave power. The functional principle of this microwave plasma source is based on a resonator with annular slot antennas (CYRANNUS^®^^2001^). This special setup allows the use of plasma from low pressure (10^−2^ mbar) to atmospheric pressure (1 bar) and above.

**Fig. 1:**
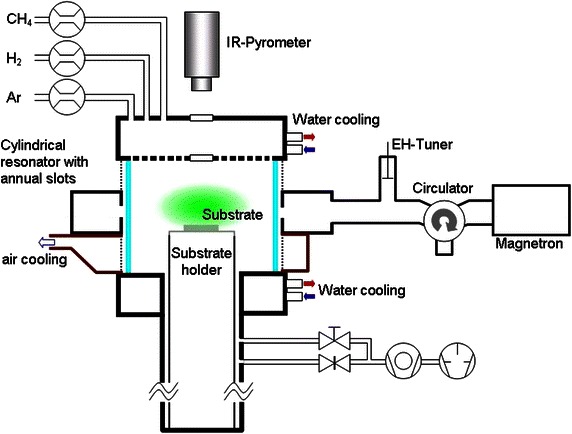
Schematic diagram of the microwave plasma source and the substrate holder showing gas inlet in *top left corner*. Due to the microwaves coupled into the resonator provided with annular slots, a plasma is ignited

The reaction chamber is a cylindrical shaped quartz tube with a diameter of 140 mm and a height of 140 mm. The process gas is fed into the process chamber by a gas shower in the top flange and a molybdenum substrate holder of 100 mm diameter is mounted on the bottom flange (Fig. 1). The substrate is placed right below the plasma.

Carbon films were deposited on 50-μm-thick copper foil (Puratronic 99.9999 %, item No.: 42972, Alfa Aesar GmbH & Co KG, Karlsruhe, Germany) similar to those described elsewhere (Li et al. 2009).

After placing the copper foil in the center of the reaction chamber the reactor was evacuated to 1 × 10^−5^ mbar. Hydrogen (200 sccm) was introduced in the chamber and a plasma was ignited at a pressure of 5 mbar with a microwave power of 1–2 kW. Plasma cleaning was executed for 20 min to clean the substrate from all remaining organic contaminations and for chemical reduction of the surface. During that time the pressure was raised. Pressure was varied between 20 and 80 mbar for the different experiments. A suitable substrate temperature for the deposition process was measured with an optical pyrometer to be 950–1030 °C. The synthesis of carbon films starts with the introduction of 1.2–36 sccm methane into the process chamber and was performed for 8 min with the methane-hydrogen plasma at constant pressure and substrate temperature. The total gas flux (i.e. hydrogen + methane) was 120 sccm during all coating processes. After that time the reactor was immediately evacuated to inhibit the etching influence of hydrogen on carbon structures at high temperatures. An overview of the process parameters can be found in Table 1.

**Table 1:** Range of relevant process parameters

Pressure	Power	Cleaning process			Synthesis		
		Time	H_2_ flow	CH_4_ flow	Time	H_2_ flow	CH_4_ flow
50–65 mbar	1 kW	20 min	200 sccm	–	8 min	118.8–84 sccm	1.2–36 sccm

Relevant process parameters and the maximum and minimum values are depicted. While the cleaning process has been held constant as well as the process time, different microwave powers, process pressures and gas flows have been applied. The total gas flow, however, has not been alternated and has been kept at a constant value of 120 sccm during the process

The PE-CVD process uses hydrogen and methane as reaction gases as is typically done in thermal CVD process for the deposition of Graphene as well as diamond. The chemical fundamentals of the synthesis of Graphene are based on a physisorption process of hydrogen and a consecutive chemisorption process of methane (Zhang et al. 2011). Physically adsorbed H_2_ molecules decompose due to the high temperature on the copper surface. Consecutively chemisorption of methane molecules results in carbon species bonded to the top layered copper atoms, which become successively dehydrogenized and eventually building the graphene’s honeycomb pattern. Due to the catalytic influence of copper and the minor solubility of carbon in copper, this leads to the growth of monolayer graphene. The graphene growth process is self-limiting and therefore basically stops after the growth of a monolayer (Li et al. 2009).

As for the synthesis of diamond it has been well established that the methyl radical is the growth precursor for CVD-diamond growth. In standard hydrogen-rich gas compositions it is produced by the abstraction of one hydrogen atom from the methane molecule via its reaction with atomic hydrogen: CH_4_ + H → CH_3_ + H_2_. In a hydrogen-deficient atmosphere—as used for UNCD growth—it is produced by electron collisional impact. This contribution leads to a non-vanishing growth rate even in hydrogen-free gas mixtures (Gruen 1999). Thus, it follows that—at constant gas composition, gas pressure, microwavepower, and substrate temperature—the plasma chemistry is fixed and the growth rate of diamond grains should be constant. There is evidence that the nucleation process for diamond differs from these reactions (Buck 2008). Since in deposition of nanocrystalline diamond films the strongest visible emission in the optical spectrum comes from the C_2_ line the C_2_ dimer was viewed as main growth and nucleation species (Gruen 1999; Gruen et al. 1994). Recently it was resolved for the first time by separate examination of the nucleation and the growth of diamond that both are consecutive but yet independent growth steps. It was shown that by controlling the C_2_/H_α_ ratio it is possible to adjust the nucleation density and the growth rate of the deposited films (Woehrl and Buck 2011). These experiments lead to the idea, that the C_2_/H_α_ ratio can also be used to adjust the deposition process between a preferential graphene and a preferential nanodiamond growth on copper substrates.

### Diagnostics

To monitor the plasma properties the optical emission spectrometer (OES) was coupled to the process chamber via a glass fiber. The entire system was calibrated with a tungsten strip lamp. The influence of the process parameters on the carbon dimer d3Π → a3Π Swan band emission at 516 nm was investigated and a correlation with the film properties (quality of the graphene, nucleation density of diamond, growth rate of diamond) was made. A linear correlation between the intensity of the C_2_-line and the actual C_2_ concentration in the plasma has been proved (Goyette et al. 1998) and therefore the measurement of a C_2_/H_α_-ratio provides quantitative information about the plasma and thus can be considered to be a reasonable measure for the plasma chemistry.

Raman spectroscopy is used for the characterization of graphene because it is a popular non-destructive analytical tool used to characterize the structure of the carbon materials (Malard et al. 2009). Raman spectroscopy is also suitable to determine the number of graphene layers, the disorder and the doping of the graphene (Ferrari et al. 2006; Casiraghi et al. 2007). The Raman spectra in this paper were measured with a Renishaw inVia REFLEX Raman spectrometer with a 532 nm (2.34 eV) and a 633 nm (1.96 eV) laser.

The Raman spectrum of graphene has a few prominent features. The G-band at around 1580 cm^−1^ arises from the *E*_2*g*_ in-plane vibration of sp^2^ carbon atoms. Two additional features, the D-band which is assigned to the A_1g_ breathing mode at the Brillouin Zone boundary K and the D′-band appear at around 1330 and 1620 cm^−1^, respectively, when point defects are introduced in the graphene layer as demonstrated in a controlled manner by ion implantation (Dresselhaus et al. 2010). Therefore I_D_/I_G_ intensity ratio can be used to quantify disorder in a graphene monolayer by determining the average distance between defects L_D_ (Lucchese et al. 2010). Moreover, for large disorder the full width at half-maximum (FWHM) of the D and the G-band is even a better measure for structural disorder (Cançado et al. 2011).

The D + D′ band (around 2920 cm^−1^) is the combination of phonons with different momenta around K and Γ and therefore provides a measure for the defect density.

The 2D band is the second order of the D band (Tuinstra and Koenig 1970; Gupta et al. 2006). Since the 2D band originates from the momentum conservation of two phonons with opposite wave vectors, no defects are required for its activation. The shape of the 2D band is of special interest because it provides information about the number of graphene layers in the material. In the case of single-layer graphene, the 2D band is fitted by a single narrow Lorentzian function. A FWHM of 33 cm^−1^ (measured with 532 nm) for the 2D band is typically assigned to defect free SLG (Stampfer et al. 2007), whereas for few-layer samples the 2D feature is becoming significantly broader and asymmetric. Bilayer graphene has a much broader and up-shifted 2D band with respect to single-layer graphene due to its special electronic structure, consisting of two conduction bands and two valence bands (Malard et al. 2009; Ferrari et al. 2006; Jorio et al. 2010). Additionally, the intensity of the G band increases with increasing layer thickness, which allows further estimation of the layer thickness (Wang et al. 2008; Ni et al. 2010).

The UNCD crystals on the surface can also be characterized by Raman spectroscopy. The main features found in the spectra of UNCD are the already described D- and G-peak due to sp^2^-bonded carbon in the film. Two additional peaks at 1150 and 1480 cm^−1^ are assigned to NCD and UNCD although their true origin is still discussed. Some authors found evidence that these peaks originate from *trans*-polyacetylene in the grain boundaries (Castiglioni et al. 2004; Ferrari and Robertson 2004; Birrell et al. 2005). Polyacetylene is an organic polymer with the repeat unit (C_2_H_2_)_n_. The polymer consists of a chain of carbon atoms with alternating single and double bonds between them, each bonded with one hydrogen atom. In this paper Raman spectroscopy is used to quantitatively identify UNCD.

Additionally SEM measurements (JSM-7500F, JEOL) are used in this work to identify the crystalline structure of the deposited diamond crystals as well as to measure the nucleation density and the growth rate of the diamond crystals.

## Results and discussion

### Influence of plasma parameters on substrate temperature

**Fig. 2:**
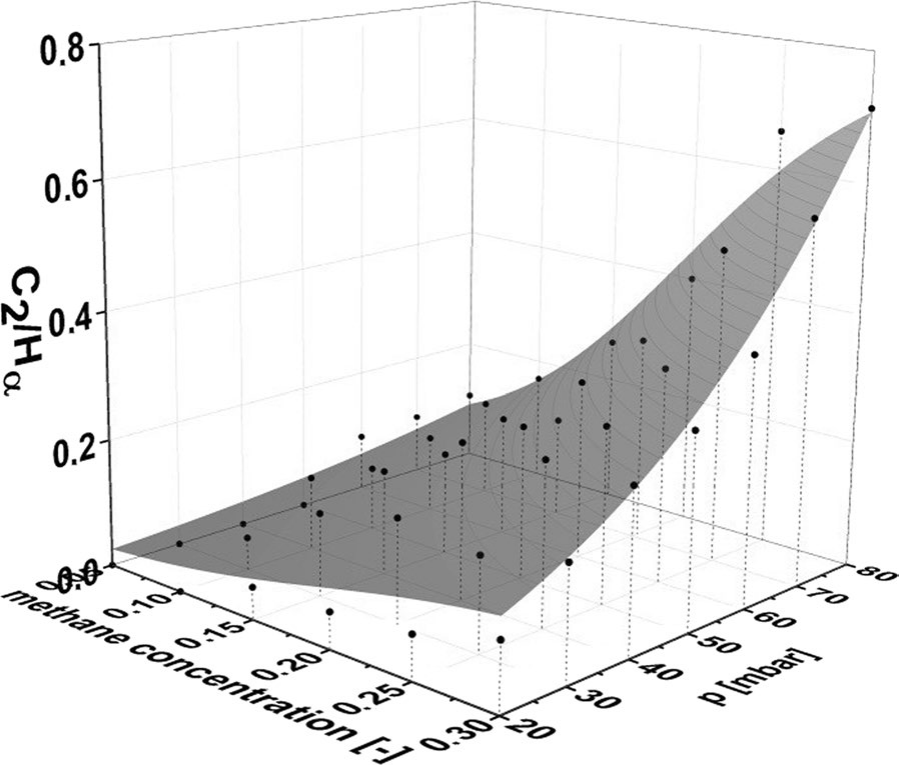
Plasma chemistry pictured in a 3D plot. 3D area showing the behavior of the C_2_/Hα-ratio as a function of the applied pressure and the methane ratio in the gas mixture. The microwave power is held at a constant value of 1 kW for all measuring points depicted. The *bullet points* are the measured values for pressures between 20 and 80 mbar and methane ratios in the gas of between 5 and 30 % respectively. The *grey leveled area* is a logistic fit of the total amount of measured values

OES is conducted to outline the influence of the macroscopic parameters pressure and methane concentration on the C_2_/H_α_-ratio in the plasma at constant microwave power 1 kW. In Fig. 2 the evolution of the C_2_/H_α_-ratio is depicted as a function of the pressure in a range from 20 to 80 mbar. In addition to that, the methane concentration in the plasma is alternated from 5 to 30 % in steps of 5 %. The measured values are shown as black spots whereas the grey levelled area is a 3D logistic fit. Both increasing pressure and increasing methane concentration lead to a strictly monotonically increasing C_2_/H_α_-ratio.

**Fig. 3:**
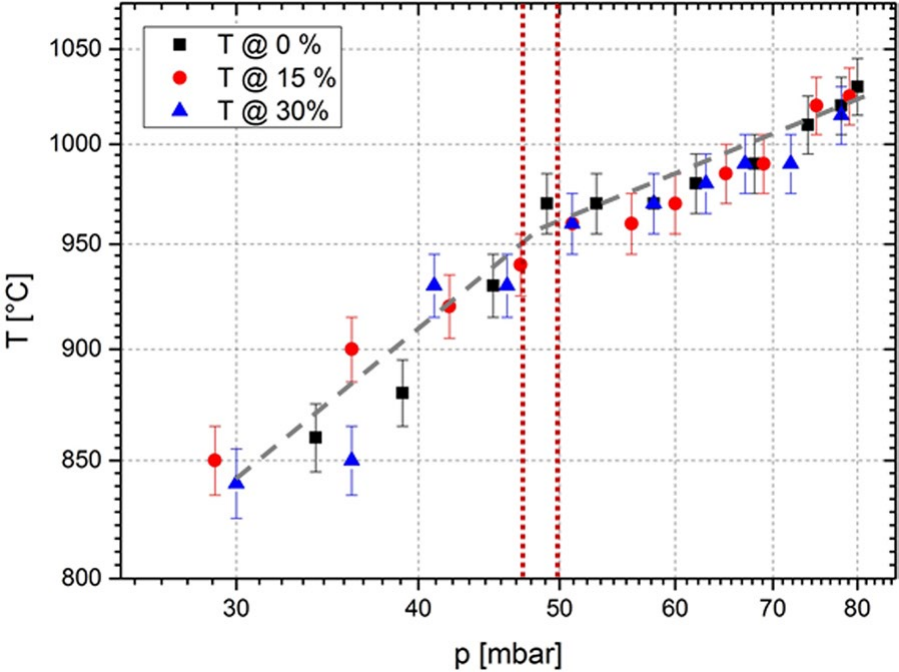
Substrate temperature as a function of the pressure at different methane concentrations. The substrate temperature for different methane concentrations is plotted as a function of the process pressure two different power laws can be identified by fitting the two regimes in a log/log-plot. The *vertical dashed lines* indicate the approximate transition region between the two regimes

As for the deposition of graphene on copper the substrate temperature has been found to have an important influence on the quality of the graphene, temperature measurements of the substrate are conducted as a function of process pressure. The substrate temperature is determined using an optical pyrometer. The general trend in this diagram is that higher pressure leads to higher substrate temperatures that originate form a denser plasma and thus more high-energetic particles interacting with the substrate. However, the two parts of the diagram shown in Fig. 3 separated by the red dashed line are found to depend on two different power laws. For small pressures the dependency is as follows

$$T \propto p^{2.5}$$

whereas for high pressures the temperature as a function of the pressure can be described as

$$T \propto p^{0.17}$$

The change in slope originates from different heating mechanisms. The higher process pressure also leads to a reduced plasma ball size. As for the higher pressure above approx. 50 mbar the plasma ball is found to lose contact to the substrate beneath. Although the substrate is still heated by the radiation originating from the plasma, direct heat transfer by energetic particles is strongly reduced. This is because the distance between the plasma and the substrate is in the range of mm and energetic particles lose their energy by collisions in the gas, since the mean free path of molecules at a pressure of several tens of mbar is only in the range of a few µm.

Repeating the measurements for different methane concentrations (0, 15 and 30 %) reveals that there is no significant influence of the methane concentration on the pressure–temperature-dependency.

### Influence of the C_2_/H_α_-ratio

As the size of the plasma ball in MW PE-CVD depends on both the process pressure and the microwave power, these two macroscopic parameters influence the substrate-plasma coupling and therefore the substrate temperature (Rau and Trafford 1990). In order to investigate the influence of the C_2_/H_α_-ratio on the quality of graphene properly, both substrate-plasma coupling and substrate temperature need to be kept constant.

Therefore experiments are conducted at constant microwave power and constant pressure using different methane concentrations in the process gas. In order to obtain a reasonable substrate temperature for the deposition of graphene on copper, 50 mbar and 1 kW microwave power are chosen. They correspond to a substrate temperature of 960 °C (for an overview of detailed process parameter see Table 2). A temperature in this range is considered to be reasonable for the deposition of graphene in a copper-catalyzed CVD process (Li et al. 2009; Srivastava et al. 2010). Since the methane concentration neither influences the substrate temperature, nor the plasma ball size significantly, the desired change of the plasma chemistry is triggered by increasing methane concentrations. Methane concentrations of 1, 14 and 27 % lead to C_2_/H_α_-ratios of 0.045, 0.14 and 0.3 respectively. The experiments are conducted providing a total gas flux of 120 sccm and a deposition time of 8 min.

**Table 2:** Process parameters applied for the experiments investigating the C_2_/H_α_-ratio influence

T (°C)	960	960	960
C_2_/H_α_	*0.3*	*0.14*	*0.045*
p (mbar)	50	50	50
P (kW)	1	1	1
Methane concentration	0.27	0.14	0.01

In italic letters the desired C_2_/H_α_-ratio is depicted. Using the information provided by the Figs. 2 and 3, sets of parameters can be found that facilitate the changes C_2_/H_α_-ratio while the plasma ball dimensions and the substrate–plasma-interaction remains unchanged

**Fig. 4:**
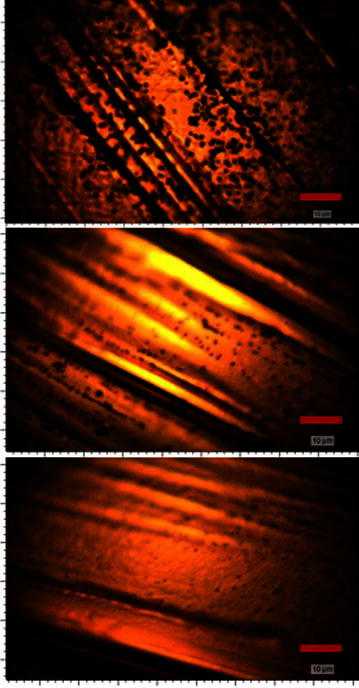
Overview photos of samples synthesized under different plasma conditions. *Left* C_2_/H_α_ = 0.3, *middle* C_2_/H_α_ = 0.14 and *right* C_2_/H_α_ = 0.045 realized by reducing the methane concentration. Photos are taken by an optical microscope using a ×100 magnification. All *scale bars* indicate a length of 10 µm. While the size of the nanocrystalline diamond spheres is proportional to the C_2_/H_α_-ratio, a higher nucleation rate can be observed as the C_2_/H_α_-ratio is reduced

**Fig. 5:**
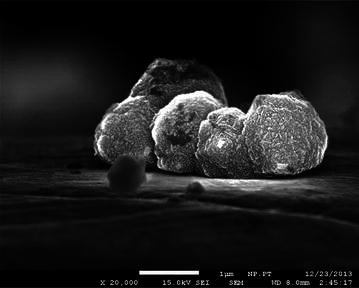
High resolution SEM image of diamond spheres. The image shows a typical accumulation of nanodiamond spheres on a graphene-covered copper surface, the length of the *scale bar* is 1 µm. Diamond facets can be observed on the* front side* of the second sphere from the* right*

The samples are investigated with respect to the quality of the deposited graphene film. Overview photos reveal that during the process black spots are deposited. It is subsequently shown by Raman spectroscopy and SEM images that these black spots are almost ball-shaped nanodiamond clusters. In Fig. 4 photos of the surfaces are presented where the C_2_/H_α_-ratio decreases from the top photo (C_2_/H_α_ = 0.3) to the bottom (C_2_/H_α_ = 0.045). All the photos are taken with a 100× magnification. An SEM image of the diamond crystals is shown in Fig. 5. Small facets on the front indicate the crystalline structure of the spheres.

**Fig. 6:**
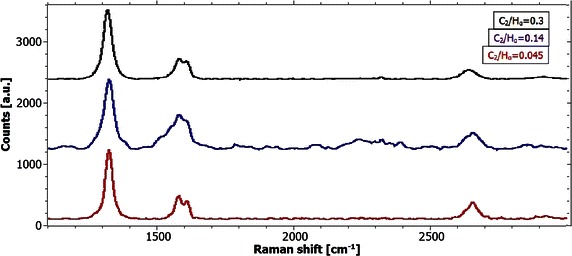
Raman spectra of graphene samples coated under different plasma conditions. Representative Raman spectra of the graphene-covered areas of the samples coated at 1 kW, 50 mbar and a methane concentration of 27 % (*black spectrum*, leading to a C_2_/H_α_-ratio of 0.3), 14 % (*blue spectrum*, C_2_/H_α_-ratio of 0.145) and 1 % (*red spectrum*, C_2_/H_α_-ratio of 0.045). Typical properties of spectra of damaged graphene are observed. Interestingly, the quality of the three graphene films is very similar in spite of the different plasma chemistry

Graphene is found in the areas between the nanodiamond clusters. Figure 6 shows one representative Raman spectrum for every sample of the area between the spots. For all Raman spectra in this chapter a background subtraction is applied. From the analysis of the Raman spectra it can be derived that the C_2_/H_α_-ratio has no decisive influence on the quality of graphene on copper substrates. On all the samples a constant I_2D_/I_G_-ratio can be found. Moreover the I_D_/I_G_-ratio is constant for all the investigated samples. The symmetric shape of the 2D-peaks is commonly considered to be a hint for monolayer graphene. However, the high intensity of the D-Peak and the shoulder of the G-Peak are strong evidences for graphene structures with high defect density. Quantitatively the slight broadening of the three characteristic peaks in combination with an I_D_/I_G_-ratio of about three is indicating an average distance between point defects of about 2 nm in the measured graphene layers (Cançado et al. 2011).

**Fig. 7:**
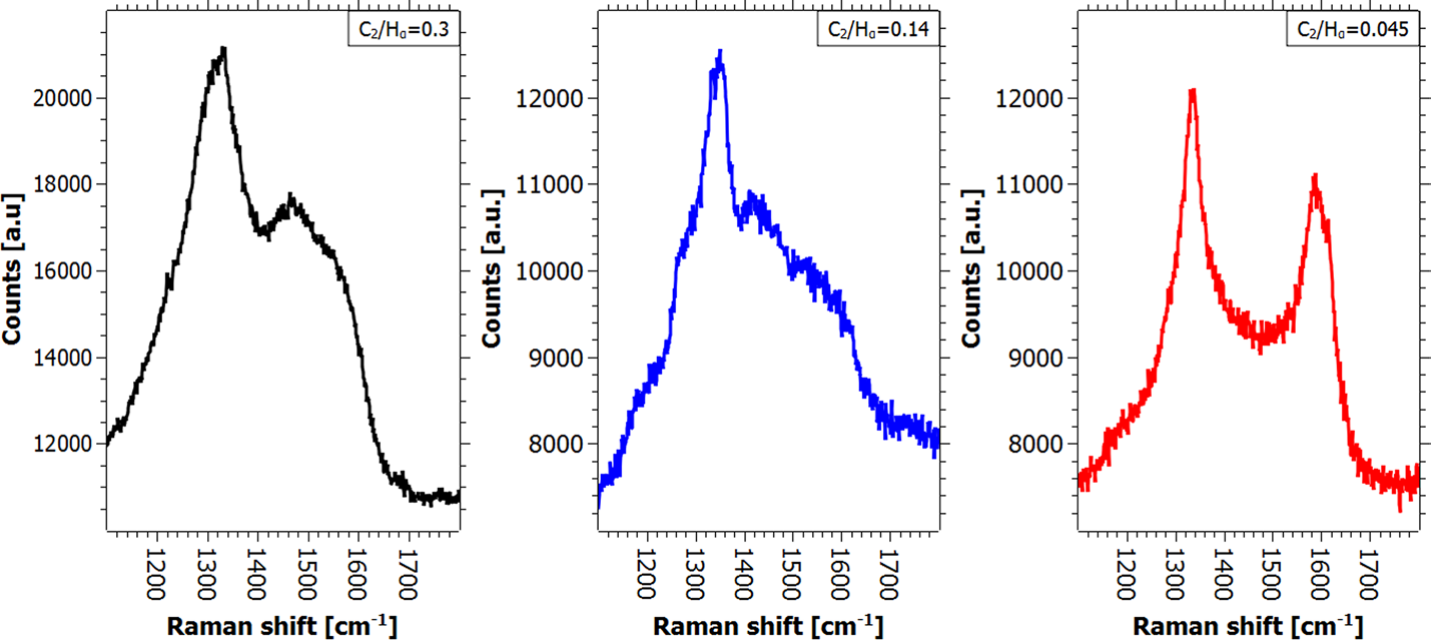
Raman spectra of the nanodiamond spheres of the samples under different plasma conditions. Representative Raman spectra of the graphene-covered areas of the samples coated at 1 kW, 50 mbar and a methane concentration of 27 % (*black spectrum*, leading to a C_2_/H_α_-ratio of 0.3), 14 % (*blue spectrum*, C_2_/H_α_-ratio of 0.145) and 1 % (*red spectrum*, C_2_/H_α_-ratio of 0.045)

In contrast to the widely unchanged graphene quality, the Raman spectra of the nanocrystalline diamond clusters seem to reveal a notable structural difference between the three samples (see Fig. 7). While the previous Raman spectra suggest the deposition of exclusively sp^2^-bonded carbon, the spectra of the diamond clusters taken between 1000 and 1800 cm^−1^ reveal the ordninary shape of nanocrystalline diamond films. The Raman spectra of all diamond clusters reveal the four most significant features of nanocrystalline diamond, namely the two broad peaks around 1335 and 1560 cm^−1^ (D- and G-Peak respectively) and the two humps located at about 1150 and 1480 cm^−1^ (Sharma et al. 2011). D and G peaks are both due to sp^2^ bonded carbon situated at the grain boundaries in NCD films. Since all spectra were taken by lasers in the visible light range only larger diamond crystals would feature a sharp line at 1332 cm^−1^ in the spectra originating from the sp^3^ bonded carbon. The results obtained from this analysis indicate that the spheres on the surface are in fact agglomerations of small nanodiamond crystals with sizes below 100 nm.

Further interpretation of the nanodiamond spheres is difficult with Raman spectroscopy because the laser spot size is notably larger than the spheres that are meant to be investigated. Consequently a high percentage of the signal originates from interactions of the laser spot with surrounding and underlying graphene.

**Fig. 8:**
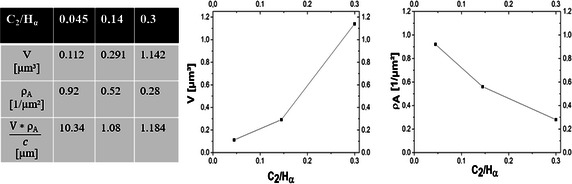
Area density and size analysis of nanodiamond spheres. Results of analysis for the sphere size and the sphere area density for increasing methane concentrations (*left*) and the graphical result for sphere size (*middle*) and the sphere’s area density (*right*) as a function of the C_2_/H_α_-ratio. An increasing C_2_/H_α_-ratio leads to taller nanodiamond spheres whereas the C_2_/H_α_-ratio is inversely proportional to the number of spheres per unit area

The volume V of the diamond clusters and the nucleation density of theses clusters ρ_A_ on the copper substrate was measured as a function of the C_2_/H_α_-ratio (Fig. 8). It is shown that the average sphere volume increases for higher C_2_/H_α_-ratios. In contrast to that the nucleation density is smaller for higher C_2_/H_α_-ratios. Based on these results it can be stated that the nucleation of diamonds on the copper surface takes place in plasmas with lower carbon content whereas the nucleation of diamond on diamond (hence the growth of the diamond clusters) requires possibly high C_2_ ratios.

The overall deposition efficiency of sp^3^-bonded carbon is estimated as the product of the volume of one sphere and their area density divided by the concentration of methane in the gas flux (see last row of the table in Fig. 8). A small coefficient indicates inefficient deposition whereas high deposition efficiencies are characterized by large coefficients. For the three experiments conducted within this series it can be concluded that the deposition efficiency of sp^3^-bonded carbon is much higher for small a methane content and thus a respectively small C_2_/H_α_-ratio. The observation of improved diamond growth in plasmas with a poor methane concentration is in good agreement with the literature (Woehrl and Buck 2007; Sharma et al. 2010).

Summarizing the results of this paragraph, the quality of the synthesized graphene does not significantly depend on the C_2_/H_α_-ratio measured in the plasma. The growth of nanodiamonds (nucleation density and diamond cluster size) which occurs in all the experiments, however, can be strongly influenced by the C_2_/H_α_-ratio. This finding suggests that the C_2_/H_α_-ratio is a process parameter that allows it to influence the growth of the different carbon allotropes independently. Moreover, the results confirm that the chemical processes that lead to nucleation and growth of graphene and diamond on copper are significantly different and that specific process parameters can be used to synthesize hybrid structures.

### Influence of the plasma-ball size

**Fig. 9:**
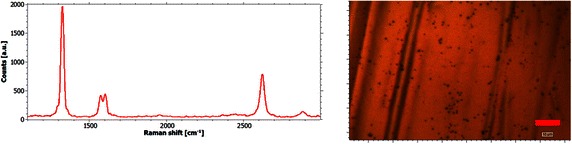
Results obtained from reduced substrate–plasma-interaction. The application of a higher process pressure (65 mbar instead of 50 mbar) leads to a compressed plasma ball and therefore reduces the plasma–substrate-interaction. *Left* Overview of surface of sample. The size of the nanodiamond spheres could be further diminished. *Right* A characteristic Raman spectrum reveals an improved film quality with respect to the defect density

Since the interaction of the substrate surface with the energetic particles from the plasma (ions, electrons) is believed to lead to point defects in the synthesized graphene layers another experiment has been conducted with a larger distance between the substrate and the plasma to reduce this influence. Therefore, the microwave power is chosen to be 1 kW at a pressure of 65 mbar leading to a substrate temperature of about 990 °C which is close to the previous process temperatures. The methane concentration was chosen to be as low as 1 % providing a C_2_/H_α_-ratio of merely 0.02. Although based on the results obtained from the previous experiments, an elevated nucleation density on the surface is expected, the nanodiamond particle growth is supposed to be widely inhibited. The results are presented in Fig. 9.

The results ought to be compared to the sample depicted in red in Fig. 6 (50 mbar, 1 kW and 1 % methane) which was synthesized at similar conditions but in direct contact with the plasma. It is found that both nucleation and growth of the nanodiamonds is reduced by enlarging the distance between the substrate and the plasma. The Raman spectrum of the sample reveals a considerably better result than the previous samples with respect to the graphene quality. From the narrow peaks and the I_D_/I_G_-ratio of approximately 4.5, a distance of 7 nm between two point defects can be derived. Therefore the average distance of point defects could be reduced by a factor of 5.3 by the larger distance between plasma and substrate.

Detailed peak analysis shows that monolayer graphene has been deposited. As there is no peak shift detected (G-peak at 1586 cm^−1^, D′-peak at 1618 cm^−1^ and 2D-peak at 2661 cm^−1^; spectrum taken at 1.96 eV laser excitation energy) there is neither considerable strain applied to the sheet, nor sheet doping can be reported.

### Influence of the direct plasma contact

It has been shown that the contact of the sample to the plasma has a negative influence on the quality of the deposited graphene. In contrast to that the direct plasma contact is found to be advantageous in order to synthesize nanodiamonds. This result is in good agreement with earlier experiments explaining the nucleation of diamond by charged or polar precursors in the plasma (Buck 2008).

**Fig. 10:**
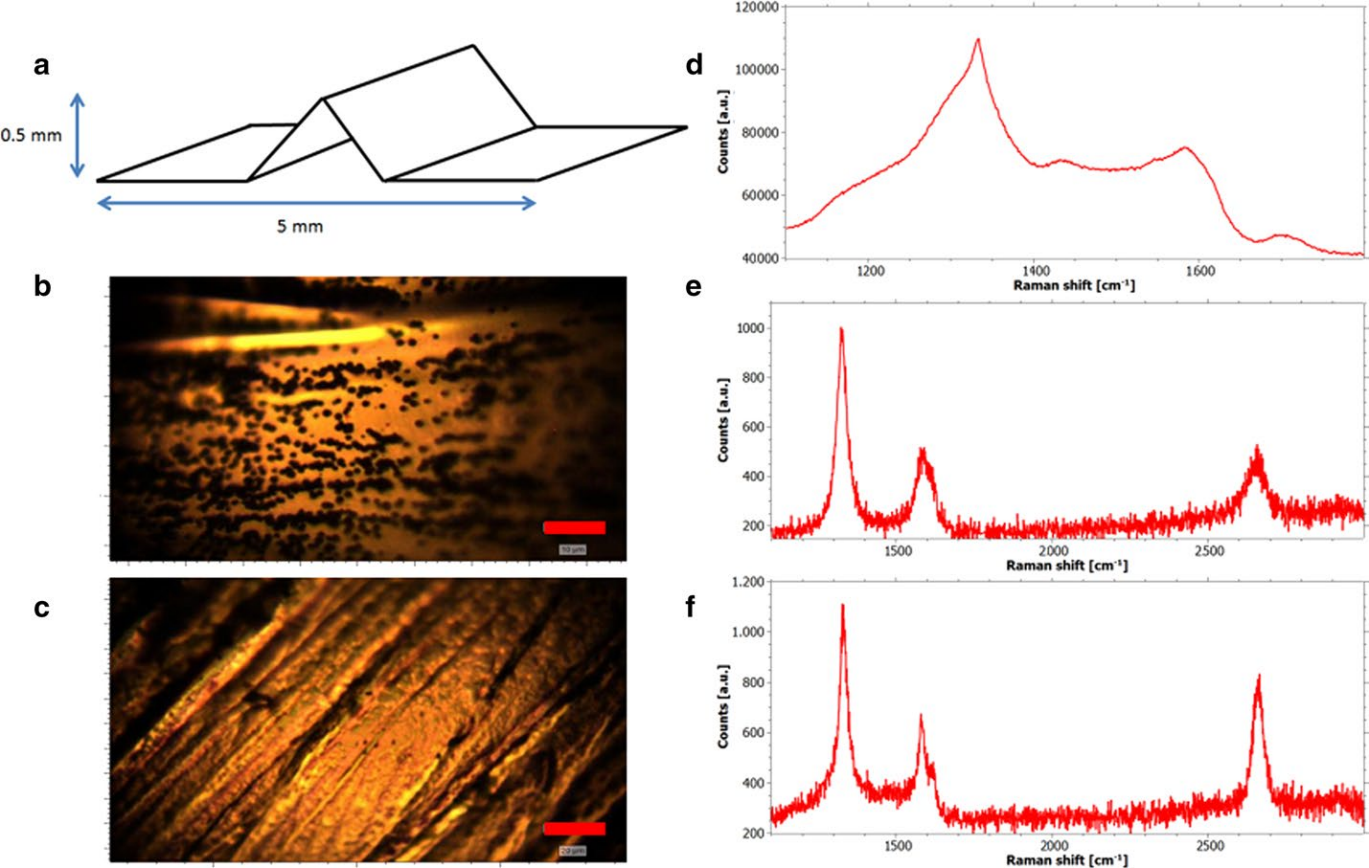
Overview of the influence of direct plasma contact. **a** The *triangular shaped* sample enables diffusive transport of species into the cavity whereas the upper side of the surface experiences a direct plasma contact. **b** Overview photo of the* upper side* of the sample: Massive nanodiamond nucleation has taken place as a result from the substrate–plasma-interaction. The length of the *scale bar* is 10 µm. **c** Overview photo of the substrate surface in the cavity. No nanodiamond nucleation is observed. Length of *scale bar*: 20 µm. **d** Raman spectrum of nanodiamond spheres on the* front side*. Characteristic diamond peaks are visible. **e** Raman spectrum of graphene between nanodiamond spheres: damaged graphene has been synthesized. **f** Raman spectrum of graphene in the cavity on the* back side* of the sample. Damaged graphene of a similar quality to the* front side* is observed. The lack of plasma interaction leads to a slightly improved result compared to the* front side*

In order to obtain additional information about the different growth mechanisms of diamond and graphene and the role the direct plasma contact has on these mechanisms a sample with a substrate geometry as shown in Fig. 10a has been fabricated.

A cavity is formed in order to enable diffusive transport to the back side of the sample, while inhibiting a direct contact to the plasma. In contrast, the upper side of the sample has direct plasma contact. Equal temperatures at the front side and at the back side of the sample are obtained in this constellation and thus the only difference between the two sides is the plasma-sample interaction.

The sample is processed at 50 mbar, 1 kW and a methane concentration of 27 % resulting in a substrate temperature of 960 °C and a C_2_/H_α_-ratio of 0.3. After the deposition the two sides of the sample are investigated separately (results see Fig. 10b–e).

Whilst strong diamond nucleation is observed at the front of the substrate, no nanodiamond can be found at its back side (see overview photos in Fig. 10b/c). Interestingly the defect density in the graphene sheet at the front side and the back side is similar. In both cases the mean distance between two point defects is estimated to be 3–4 nm. This can lead to the conclusion that most of the point defects in the graphene are not due to plasma surface interactions but due to the high temperature of the copper and the resulting evaporation during the growth.

The most important outcome, however, is that evidence of the different growth mechanisms of diamond and graphene is shown. Direct plasma contact is inevitable for the growth of diamonds. In contrast to that the growth of graphene is based on the temperature activated catalytic influence of copper which does not require direct plasma contact.

## Conclusions

In this paper the simultaneous growth of nanodiamond clusters and graphene on copper by PE-CVD process is described for the first time. It is found that the C_2_/H_α_-ratio in the plasma is a process parameter that allows to influence the growth of the different carbon allotropes independently. While the diamond nucleation can be influenced by the C_2_/H_α_-ratio, the graphene quality remains mostly unchanged by this parameter. Moreover, it is derived from the experimental data that the direct plasma contact with the copper surface is beneficial for the nucleation of the diamond clusters while the growth and quality of the graphene benefit from a larger distance to the plasma. Therefore, this work identifies crucial parameters to establish a method to deposit graphene–diamond hybrid films using a MW PE-CVD process, tailor the composition of these films, or entirely oppress the diamond deposition if desired.

## Abbreviations

MW PE-CVD: microwave plasma enhanced chemical vapor deposition
OES: optical emission spectroscopy
SLG: single layer graphene
NCD: nanocrystalline diamond
UNCD: ultra nanocrystalline diamond
FWHM: full width half maximum
